# Consensus Recommendations for the Safe Handling of Cytotoxic Agents in Cytotoxic Academic Research Laboratories (CARL)

**DOI:** 10.1177/1078155220951869

**Published:** 2020-10-14

**Authors:** Shereen Nabhani Gebara, Stephen Barton, Ian Appleford, Pauline McCalla, Graham Sewell, Racha Sabbagh Dit Hawasli

**Affiliations:** 1 School of Pharmacy and Chemistry, Kingston University, Kingston Upon Thames, UK; 2 Pharmacy Department, London North West Healthcare NHS Trust, London, UK; 3 Leicester School of Pharmacy, De Montfort University, Leicester, UK

**Keywords:** Cytotoxic Agents, Academic Research Laboratory, occupational exposure, academic researchers, guidelines

## Abstract

Cytotoxic agents, also called antineoplastic agents, are used in cancer treatment due to their inherent activity to inhibit cell growth or proliferation, or DNA, RNA and protein synthesis. They are, therefore, hazardous by nature in a non-selective manner leading to disruption of cell growth and function of both diseased and healthy cells of treated patients.

While the benefits of receiving cytotoxic agents may outweigh the incurred risks for patients, the same cannot be said for exposed healthcare practitioners involved in the transport, preparation, administration, and resulting waste disposal of these agents.

Consequently, many professional bodies around the world have set standards of practice to prevent occupational exposure of healthcare workers to cytotoxic agents, and hospitals have been active in defining strict policies in this concern.

However, due to the variability of the practice and infrastructure in academic settings, some activities performed within the cytotoxic academic research laboratory often do not adhere to recommendations published by guidelines.

The present recommendations were therefore set forward by members of a working group who are experts on the subject matter representing academic, clinical, and research backgrounds in an attempt to promote safe cytotoxic handling in academic institutions.

The document maps out the trajectory of cytotoxic agents being investigated in academic research laboratories while providing recommendations on the delivery, storage, use and disposal of cytotoxic agents in university settings.

## Abbreviations

ACDP: Advisory Committee on Dangerous Pathogens

BSC: Biological Safety Cabinet

CA: Cytotoxic Agent

CAs: Cytotoxic Agents

CARL: Cytotoxic Academic Research Laboratory

LLOD: Lower limit of detection

MSC: Microbiological Safety Cabinet

NIOSH: National Institute for Occupational Safety and Health

## Introduction

Cytotoxic agents (CAs), also called antineoplastic agents, are used in cancer treatment due to their inherent activity to inhibit cell growth or proliferation, or DNA, RNA and protein synthesis. They are, therefore, hazardous by nature in a non-selective manner leading to disruption of cell growth and function of both diseased and healthy cells of treated patients.^1^

While the benefits of receiving CAs may outweigh the incurred risks for patients, the same cannot be said for exposed healthcare practitioners involved in the transport, preparation, administration, and resulting waste disposal of these agents.

Consequently, many professional bodies around the world have set standards of practice to prevent occupational exposure of healthcare workers to CAs, and hospitals have been active in defining strict policies in this concern.

However, due to the variability of the practice and infrastructure in academic settings, some activities performed within the cytotoxic academic research laboratory (CARL) do not adhere to recommendations published by guidelines.

This was highlighted in a national survey gauging current practices of cytotoxic handling within CARLs in the United Kingdom.^2^

Results revealed that due to the lack of CARL tailored guidelines, many safety considerations are overlooked.

The present recommendations were therefore set forward by members of a working group who are experts on the subject matter representing academic, clinical, and research backgrounds in an attempt to promote safe cytotoxic handling in academic institutions.

It must be noted that these recommendations should be followed alongside the policies and procedures of the organization and the national and/or federal ones in place.

## Scope

This document is aimed at

- Safety officers, laboratory managers, cleaning supervisors, waste disposal personnel, store reception, and goods in (also called Procurement) managers working in universities where research involving cytotoxic or potentially cytotoxic agents is carried out
- Research supervisors, researchers, and students involved in handling cytotoxic or potentially cytotoxic agents.

Oncology is a research-driven specialty whereby understanding the science underpinning practice and investigating interventions to optimise this practice advance the field. A significant proportion of oncology research is conducted in academic research settings and this can vary from cancer drug discovery, formulation research to practice research.

Thus, the handling of CA in academia is frequent and vital for supporting advances in cancer care.

Researchers in academic institutions often conduct analytical or animal testing on CAs to enhance their clinical use or suggest new treatment patterns. Additionally, the synthesis of compounds with potential cytotoxic activities often takes place in chemistry labs of academic institutions. All these research activities are taking place in laboratories not dedicated nor engineered for cytotoxic handling.

The document maps out the trajectory of CAs and potentially CAs investigated in academic research laboratories while providing recommendations covering the gaps related to the safe delivery, storage, use and disposal of CAs in university settings. This document does not aim to provide specific recommendations on the type of personal protective equipment (PPE), biological safety cabinets (BSC), and other items used in the handling of CAs. Reference to updated guidelines from national and international societies should be sought for this purpose (e.g. ASHP, ISOPP).

## Recommendation development method

The working group met on a quarterly basis; first, the framework of the guidance was set during introductory meetings, then members were divided into task-orientated sub-groups that addressed specific elements of the proposed recommendation. Resources were shared between members to facilitate access to published guidance on cytotoxic drug handling. Finally, preliminary drafts were submitted and discussed for consensus.

## Glossary

The definitions used in this guidance have been adopted from national and international organizations where available. Otherwise, the authors have developed their own definitions as listed below

1. Accident: an event that results in injury or ill health ^3^
2. Audit: an in-depth review of an organisation’s safety management structure and operational procedures
3. Could: used for providing suggestions from expert opinion
4.Closed system drug-transfer device (CSTD) as “a drug transfer device that mechanically prohibits the transfer of environmental contaminants into the system and the escape of hazardous drug or vapour concentrations outside the system” ^4^
5. CARL is defined as any laboratory in a university or research centre where cytotoxic agents, classified as hazardous, are manipulated, temporarily or permanently, to investigate and enhance drug properties.
  a. A dedicated CARL is defined as a specialist laboratory where only CAs are investigated.
  b. A temporary CARL is defined as a general-purpose laboratory with temporary use of equipment within for CA investigation.

Examples of temporary CARLs include but are not restricted to

- *Chemistry research laboratories* that are sometimes used for the synthesis of new compounds with potential cytotoxic activities. These compounds should be treated as cytotoxic until proven otherwise as dictated by the National Institute for Occupational Safety and Health (NIOSH) definition of hazardous drugs^5^
- *Animal testing laboratories*

This guidance is limited in targeting the laboratories for animal testing as there are specific guidances that target this matter within its scope ^6,7^

Where cytotoxic agents are tested on animals held in cages, special attention should be addressed to the excrement and carcasses. These need to be treated as cytotoxic waste. Other safety issues, such as the controlled laboratory access, PPE, waste disposal, etc. should be addressed as dictated in this guidance. When the referenced guidance on animal testing above and the present one target the same matter, the more vigilant guidance overrides.

(iii) *Cell culture laboratories*
(iv) *Instrumentation laboratories*; containing HPLC, NMR, etc.

Activities undertaken in these laboratories may include but are not restricted to the following: synthesis of potentially cytotoxic compounds, storage of CA vials and ampoules, reconstitution of CA, dilution of CA from concentrated solutions, transferring CA into tubes or glass vials for chemical analysis, running CA samples in analytical chemistry equipment, animal testing with CA, animal retention in cages, cage wash facilities, etc.

6. Cytotoxic agents: “all drugs with direct anti-tumour activity, including conventional anti-cancer agents, monoclonal antibodies and partially targeted agents (e.g. kinase inhibitors, such as sunitinib, imatinib), and drugs such as thalidomide and lenalidomide.” ^8^

Cytotoxic agents are classified as hazardous which is defined by the NIOSH^5^ as *“*those that exhibit one or more of the following six characteristics in humans or animals:

• Carcinogenicity
• Teratogenicity or other developmental toxicity
• Reproductive toxicity
• Organ toxicity at low doses
• Genotoxicity
• Structure and toxicity profiles of new drugs that mimic existing drugs determined hazardous by the above criteria”
7. Detergent=cleaning agent with wetting agent and emulsifying-agent (tensio active) properties.
8. Goods in: it is the department which handles, delivers, distributes, and processes the goods and parcels delivered to the academic organization
9. General store refers to the storage section in the academic organization
10. Incident:
  a. near miss: an event not causing harm, but has the potential to cause injury or ill health ^3^
  b. undesired circumstance: a set of conditions or circumstances that have the potential to cause injury or ill health,^3^ e.g. untrained personnel handling CA
11. Laboratory Containment levels

The Advisory Committee on Dangerous Pathogens (ACDP) have set three laboratory containment levels to ensure the safety of the workers and community in all research, teaching, clinical, forensic, veterinary and environmental organizations.^9^

A CL1 laboratory is suitable for secondary education and undergraduate teaching where biological agents unlikely to cause disease in healthy humans are handled.

A CL2 laboratory is assigned for clinical, diagnostic and research work involving low to moderate risk biological agents, those with accessible effective prophylaxis and treatment, and unlikely to be transmitted.

The highest containment level is a CL3 laboratory offering satisfactory protection to workers and other members of the community from the direct effect or transmission of hazardous biological agents.

12. Should: used to say or ask what is correct or best thing to do.^10^ It is used to show an obligation.

## Recommendation sections

## 1. Goods in

When ordering a CA, the researcher should make sure the delivery package is delivered only through goods in of the relevant organization and not bypass it regardless of how small the quantity is.

### 1.1. Reception of CAs in goods in department:

*1.1.1.* The researcher should inform the goods in and the store when expecting to receive a CA. Not all CA packages received are usually labelled with a hazard caution sign; however, requesting the sender to label it might fall outside of the requirements of labelling. Hence, the procurement at the university could agree with the supplier to label the packaging accordingly.

*1.1.2.* The staff at goods in should be aware that they are receiving a CA and should be briefed on their possible hazards. In particular, it should be recorded that the package was not physically damaged upon reception. In case of evidence of physical damage on the parcel, it should be accepted from the courier to avoid exposing the driver to cytotoxic hazards, and the safety officer should be notified. The damaged parcel should be treated as a spill and disposed of as per guidelines.

A risk assessment should be in place for the storage of CAs including emergency procedures, which the goods in and store staff should be familiar with.

*1.1.3. Records of CA: *Records indicating the below should be maintained:

- The time and date of parcel receipt
- The name of the company delivering the CA
- The staff who accepted the delivery
- The type and amount of CA delivered
- The date of pick up by the researcher
- The physical state of the received package

This serves as a tracking system for occupational exposure as well as for CA flow within university premises.

Universities could consider having a stock tracking system in place; this is a program that will track the use and users of all CAs received and handled within university premises in order to: (1) record researcher exposure, (2) track consumption and depletion of the CA, and (3) facilitate acquisition of the CA by other researchers working on the same molecule.

#### 1.2. Storage

CAs should be stored in a separate zone within the storage area away from traffic, within a dedicated refrigerator or cabinet securely locked with limited access.

A mechanical digital lock should be considered to lock the cabinet or refrigerator as the digits don’t become discernible after repeated use as is the case with electronic digital locks.

#### 1.3. Qualification/training

Store reception and goods in staff should have a basic knowledge of the hazardous properties of CAs and receive training on the use of the spill kit

#### 1.4. PPE

The staff should wear adequate PPE when handling parcels with visible signs of damage/spill. The staff could be advised to wear PPE when handling parcels that haven’t lost their integrity as a precautionary measure.

### 1.5. Spill kit

An appropriate spill kit should be present in the store near the CAs storage zone and should be used in case of a spill or if there is sign of spill on the outer packaging (refer to section 2.1 on spill kit content).

The store staff should not handle spillage or leakage, rather they should post a sign to warn people not to go near the contaminated area. Additionally, they should know whom to contact and the area should be evacuated if necessary.

The chemical safety officer or member of the departmental safety team dealing with spillage and leakage should be notified to deal with this event.

#### 1.6. Incident reporting system

Classification: There should be an incident reporting system in place, and it should comply with the organisation's reporting process.

## 2. Transport

Within university premises; intact package(s) picked up from ‘goods in’ could be transported to the laboratory as they are, there is no need for a lab coat or PPE

### 2.1. Spill kits

A cytotoxic spill kit should be carried during transportation of CA vials and tubes within university premises.

The cytotoxic spill kit should enable quick and safe access to remove commonly listed/used cytotoxics and should comply with the requirements set forth by recognized guidelines such as AJHP or ISOPP.

The spill kit should contain the following items at the least^7^

- User's instruction
- Warning signs
- Personal protective equipment: Impermeable protective gown, shoe-cover, head-cover, goggles/face shields, respirator mask, Chemotherapy gloves
- Swabs
- Concentrated alkaline detergent solution
- Clearly labelled cytotoxic waste container.
- Spill report/incident form.

### 2.2. Leak proof containers

Leak proof solid containers with visible yellow hazard labelling should be used for transporting cytotoxic materials contained within test tubes or other containers (e.g. HPLC vials) within university premises. A leak proof bag could be used as an alternative.

## 3. CARL

### 3.1. Classification

ACDP CL2 is a minimum requirement for laboratories where CAs are handled.

### 3.2. Access

#### 3.2.1. Signage

Appropriate signage should be clearly posted on the external side of the entrance door to indicate in non-scientific wording the type of exposure within the laboratory. Moreover, the sign should also highlight the fact that the CARL has restricted access for that reason.

#### 3.2.2. Restricted access

CARL access should be restricted to authorised personnel. This could be implemented using access control. Moreover, any cleaning or maintenance work should not be performed without at least notifying (if not the presence) of the laboratory manager or authorised technical staff. Cleaners and maintenance staff should also go through induction before accessing. Maintenance managers should liaise with laboratory manager. There should be a process that protects the integrity of the laboratory, equipment and the safety of the staff.

#### 3.2.3. Secure storage of cytotoxic agents

Cytotoxic agents used within an institution should only be stored within a dedicated CARL. Within the latter, there should be a dedicated locked cabinet and/or fridge for storing opened/unopened vials. Similarly, this applies to prepared stock solutions and samples that could be stored in a separate cabinet or fridge.

Except for unopened vials, everything should be clearly labelled with date of preparation, date of opening, name of cytotoxic agent, concentration and initials of researcher.

### 3.3. Cleaning and maintenance works within CARL

#### 3.3.1. Personnel:

Cleaners should be trained and supervised to perform cleaning.

Engineers should be provided with an induction session.

For safety of the staff and integrity of the laboratory, access to CARL should be sought before being accessed by cleaning and maintenance staff.

#### 3.3.2. PPE

The cleaning staff should use safety glasses/appropriate eye protection or face shield (if possibility of splashing), protective chemotherapy gloves, overshoes, and disposable gowns to protect their own clothes. They should wash their hands thoroughly with soap and water immediately after removing the gloves.

#### 3.3.3. Cleaning utensils

Dedicated cleaning utensils (e.g., wipers, mops, and disinfectants) for use in the cleanroom should be made of materials that generate a low amount of particles. All cleaning tools should be non-shedding and dedicated to use in the laboratory.^11^ A mop could be used and discarded as cytotoxic waste, as an alternative, a disposable head/wipe could be used to avoid worrying about storing of these dedicated utensils.

If cleaning tools are reused, their cleanliness should be maintained by thorough cleaning and disinfection after use and by storing in a clean environment between uses. These should not be shared with other laboratories.

#### 3.3.4. Cleaning methodology

 Researchers are responsible for cleaning the workbenches and surfaces. Cleaners are responsible for cleaning the floor. Cleaning should take place when no active experimentation is taking place. The laboratory manager should keep a log of cleaning activities.

Cleaning should proceed from the cleanest area to the dirtiest area of the room. This would involve a ceiling to floor cleaning flow, moving outward from the ventilation tool to the exit.

BSC: Wipe the surface of the BSC including front, sides and bottom in the direction of the groove of the surface. Wiping should be in a continuous motion. When a corner is met, the researcher should make an ‘S’ curve and return to the opposite side while overlapping the previous stroke. The researcher should continue with fixtures (e.g., gas or vacuum valves, bar and hooks, if present), the sides, and finally the work surface.

#### 3.3.5. Approved detergents

Agents, approved by the institution, should be used to deactivate cytotoxics.

#### 3.3.6. Cleaning frequency

As per international guidelines, equipment such as the BSC as well as work benches should be cleaned after each use, and as such, left clean. A comprehensive cleaning of the laboratory should be scheduled periodically by the laboratory manager.

#### 3.3.7. Disposing of contaminated waste

Solid waste generated throughout cleaning or decontamination procedures should be collected in suitable plastic bags, sealed inside the ventilation tool, and removed with minimal agitation. Cleaning solution used to clean the floors could be discarded in the regular drains.

Liquid waste, such as that collected from test tubes, and HPLC vials, should be collected in large glass waste bottles, as they are impervious to most chemicals. Care should be taken while handling the bottle as it presents a breakage hazard, hence plastic (e.g., polyethylene jerry cans) or metal (galvanized or stainless steel) safety containers could be used.

The content and concentrations of liquid waste should be clearly and safely labelled on the bottle.

Solid and liquid waste should be accumulated in the CARL pending transfer to the institution's central facility for chemical waste handling

#### 3.3.8. Safety cabinet checks/service

Containment during changing filters: laboratory access should only be granted when no active experiment is taking place

Documentation of education, training, exposure and cleaning should be maintained

Engineers, carpenters and maintenance staff should follow the same procedure as the cleaning personnel in what concerns CARL access and PPE.

PPE comprising of shoe cover and gloves should be used at all times

Replaced filters should be disposed of as contaminated waste

### 3.4. Laboratory rules

For each CARL, standard operating procedures (SOP) should be developed and updated periodically. Within one institution, if there is more than one CARL, then a separate SOP should be developed for each CARL customised to the type of agents, experiments, and equipment used. However, the laboratory SOP should adhere to the institution’s general guidance framework, which might translate into common sections within each SOP.

For example, common sections could include education and training of researchers, reporting procedures, record of employee exposure, environmental controls, cleaning, personal contamination procedure etc.

### 3.5. Equipment

#### 3.5.1. Engineering controls

Appropriate engineering controls related to the type of investigations conducted (such as Microbiological Safety Cabinet (MSC), fume hoods, isolators) should be used in the CARL

#### 3.5.2. Maintenance and service

The engineering controls used should be well maintained and serviced to ensure safe and effective use.

#### 3.5.3. Closed system drug-transfer devices

Closed system drug-transfer devices could be used when possible

## 4. Laboratories other than CARL used for cytotoxic research; temporary CARL

### 4.1. Rationale

*Cytotoxic research:* all testing with CAs should be performed in dedicated CARL, unless major equipment is housed in other facilities (section D, definition 5-b)

### 4.2. Label

A clearly labelled, portable cytotoxic spill kit should be available wherever CAs are being prepared, stored, analysed and investigated.

### 4.3. Signage

Proper temporary signage informing all researchers of the presence of CAs and their hazards should be displayed in highly visible locations and on the external door while work is taking place. The warning should be communicated in language easily understood by all researchers (e.g. agent toxic to human cells in use).

This signage should specify the date, time and duration of the experiment, and should be removed at the end of the experiment.

### 4.4. Work mats

Work mats, preferably with an under layer to guarantee non-slip stability on work surfaces, should be used in temporary CARLs for adequate absorption and containment of cytotoxic spills

### 4.5. Cleaning

#### 4.5.1. Instruments

A clear ‘Instrument-specific’ cleaning regime should be devised before use.

For instruments where parts are in direct contact with the drug (e.g. HPLC), there should be a more rigorous regime than those where the sample is sealed (e.g. NMR).

For HPLC and LC-MS, columns and tubing should be flushed through with deionised water for an appropriate period after use.

Injection ports should be ‘activated’ five times using deionised water.

Buttons, keyboards etc. should ideally be covered with an appropriate overlay during use. If this is not possible, they should be wiped thoroughly afterwards. A keyboard protector readily available on the market could also be used. As an alternative, a transparent plastic bag could be used and later disposed of as cytotoxic waste after each use.

A separate eluent waste bottle labelled as cytotoxic should be used and removed after the work is completed.

#### 4.5.2. Workbenches

The immediate laboratory work area should be cleaned with water and detergent soap (not disinfectant) when the experimental procedures are completed for the day, by the researcher him/herself. The laboratory area where cytotoxic agents are used should not be utilized for any other purpose until cleaning has been performed. Full PPE, as mentioned in section 5.3, should be used while cleaning the area, and full PPE worn while running the experiment.

## 5. Researcher

### 5.1. Eligibility

#### 

Notice of occupational exposure and adequate training (background, theoretical training, and practical training): All researchers involved in cytotoxic preparation should be educated about the risks associated with cytotoxic handling, and of the safe handling of CA and related waste.

Exclusions from work in the cytotoxic laboratory/risk assessment

#### Illness

The Medical team should be informed and a decision on whether to carry on with this work should be made after consideration of all possible risks (e.g. in case the researcher is on long term medicines that are carcinogenic).

Family planning for male and female researchers

Pregnancy, lactation

#### Medical examination

there are no current medical/laboratory indicators robust enough to be used in assessing the occupational exposure to CAs.

### 5.2. Training

#### 5.2.1. Safe handling procedures

All researchers involved in cytotoxic preparation should have completed a certified training conforming to the outlined sections/regulations. A person designated by the institution should provide the standardized training; all education provided should be documented and related records retained.

A competency assessment of practice should be performed on a yearly basis to verify compliance with procedures (continuous professional development/annual update); the assessment could be brief but different each year.

#### 5.2.2. Content of training

The training should cover the below points as a minimum requirement

- Safety cabinet/isolator
- Types of acceptable safety cabinets
- Proper use of safety cabinets
- Working in the laboratory (check laboratory etiquette in section 3- CARL)
- Use of PPE
- Handing cytotoxic waste
- Handling cytotoxic spill
- Cleaning procedures (after each work session and in case of a spill)
- Manipulating cytotoxic vials
- Possible contamination on the outside of vials
- Withdrawing from cytotoxic drug vials

### 5.3. PPE

Types of PPE suitable for this area:

#### 5.3.1. Gloves

Gloves should not be worn for more than an hour and should be replaced immediately if there is a visible tear or leak.^12,13^

Latex, nitrile or neoprene gloves could be used as long as they meet the ASTM D6978-05 standards.^6^ Double gloves could be used when chemotherapy gloves are not used.

Gloves should be taken off safely and discarded.^6,7^

When double gloves are worn, the inner pair should be kept on to handle the analytical machinery (HPLC, etc.).

#### 5.3.2. Masks

Common surgical masks offer no protection against aerosols, and N95 respirators provide no protection against gases of vapors; they both only act as physical barrier against splashes and droplets hitting the nose and mouth of the person. Surgical masks and N95 respirators are hence both not recommended for use.^6^

Relevant guidelines need to be reviewed to check whether the type of cabinet used warrants to wear a mask or not.

A NIOSH approved disposable respirator mask (an elastomeric half-mask with a multigas cartridge and P100 particulate filter) should be used in case of a spill or when cleaning the safety cabinet.^6,7^

#### 5.3.3. Gowns

The gown used should have the following characteristics: Long and closed at the neck - Long sleeves with cuffs gripped at the wrist - Waterproof material for the front and sleeves.^6,7,12^

A dedicated gown for CARL, distinct from gowns used in other laboratories should be used.

Disposable over sleeve to protect the wrist and lower arm should be used.

If an over sleeve and a plastic apron over the gown are used and discarded after each manipulation, the gown could be kept for further use.

The gown could be folded inside out and stored in a plastic bag for further use.

White coats used in cytotoxic laboratories should be hung horizontally to avoid cross contamination that could result from hanging them on top of each other. Each gown should be labelled with researcher’s name to avoid the wrong coat being used.

University protocol or policy for washing lab coats used during experiments with cytotoxic agents should be followed (use of dedicated washrooms, dissolvable bags, etc.) otherwise, the lab coats should be discarded as cytotoxic waste.

#### 5.3.4. Eye protection/face shields

Traditional safety laboratory goggles should be used when in the laboratories and offer acceptable protection.^6,7^ A face shield should only be worn for managing spills.

#### 5.3.5. Overshoes

Overshoes should be donned as soon as the researcher enters the laboratory and should be removed second last, right before removing the inner pair of gloves, considered clean

#### 5.3.6. Work mats

A work mat should be used in areas where manipulation of cytotoxic material is taking place, i.e. reconstitution within the BSC, vials/tubes/syringes containing CA to ensure containment of contamination.

The work mat should have 3 layers of absorbing material on the top and a non-slip under layer.

#### 5.3.7. Over sleeves

Over sleeves should be worn during manipulation of cytotoxic material to minimise contamination of gowns.^12,13^ These should be (1) impermeable to commonly listed cytotoxic drugs, (2) allow comfortable manipulation, (3) have knitted cuffs for added safety and to allow extending the outer pair of gloves over the sleeves.

Using over sleeves helps reduce cost as the researcher will be able to re-use the gown rather than discarding it after each reconstitution.

#### 5.3.8. Plastic aprons

These are usually polyethylene^12^ and should provide a protective barrier to accidental spills or sprays

#### 5.3.9. Removing PPE

Over sleeves should be rolled down so that they are inside out when taken off, thus containing any contamination on the inside.

When removing the outer gloves, the exterior cuff of the first glove should be held and rolled down so to become inverted inside out. While holding that removed glove in the double-gloved hand, the second glove should be rolled down and inverted inside out over the first glove.

Gloves should be removed by touching the inside of the cuff to avoid any contamination of the inner gloves.

All used PPE should be discarded as cytotoxic waste.

#### 5.3.10. Re-using PPE

Only the white coat could be reused if “clean”, i.e. no visible contamination after the use of over sleeves and plastic apron.

#### 5.3.11. Washing facility for emergency management of splash/spill

Eye bath and means of washing should be available at the CARL or temporary CARL where CAs are handled. Anyone accessing the CARL or temporary CARL should be advised to use the closest bathroom if not available within the laboratory where they are operating. Normal soap and water could be used to wash

## 6. Occupational health

Universities could adapt the requirement of The Higher Educational Occupational Physicians/Practitioners on securing access to a specialist in occupational medicine.^14^

### 6.1. Records

Records of employee exposure researching CAs should be kept

### 6.2. Personal exposure monitoring

The standard practice dictates that there is no need for health surveillance as long as the subject is not showing any symptoms.

Routine surveillance is not evidence based, however, a log of exposure during manipulation should be kept for submission to occupational health (for health surveillance of chronic exposure) when needed.

In case of any spill or exposure, then appropriate medical attention should be sought, and the incident should be reported.

Any incident related to a cytotoxic or potentially cytotoxic compound should be logged.

## 7. Environmental controls

### 7.1. Cleaning

By definition cytotoxic drugs interrupt cellular processes and thus will be harmful to organisms and in particular the aquatic environment.

A number of simple rules should be followed when dealing with cytotoxic drug waste.

- Liquid waste/Water used for cleaning (considered contaminated) could be discarded into the sewage as it is diluted and deactivated by addition of bleach
- Liquid waste should be held in drums labelled appropriately and removed using a licensed contractor
- Solid waste should be disposed of as “Hazardous waste” and sharps disposed of in a sharps’ container with the colour of the lid highlighting the cytotoxic nature of the contents.
- The disposal of cytotoxic drug waste should be outsourced to a company specialised in the disposal of hazardous waste
- CA waste should not be mixed with any non-hazardous waste
- Removal from the site should be through a licensed contractor.
- Labels used for such waste should be identified with the European Waste Catalogue (EWC) codes of 18 01 03 or 18 01 08
- A cytotoxic drug trail from laboratory to contractor should be in place to ensure that the correct procedures are being followed
- Records could be maintained of how CAs are disposed of from the laboratory and how much is being removed over a set time period such as a week/month etc.
- Liquid spillages should be absorbed using tissues, cloths etc. Residual material should be diluted with copious amounts of detergent.
- Appropriate PPE including double gloves, laboratory coat and protective plastic apron, face shield (preferred to eye protection) should be worn when dealing with a spillage irrespective of volume or amount.
- Records of occupational exposure should be kept for up to 10 years. They should be electronic and stored in a searchable and future proof format.

### 7.2. Transport of CA within university

Transport of CA within university (e.g. between laboratories) should be carried while wearing a lab coat, holding a spill kit, and with CA contained in a leakproof container

### 7.3. Transport of cytotoxics off university premises

The authorization for carrying cytotoxics in public transport depends on the company and should be checked accordingly (e.g. not allowed on Transport for London).

The packaging should be well labelled, and the researcher should follow the same packaging principles as those described for transport within university premises (Section E- 2.Transport). Only a leak proof container and spill kit should be used.

Whenever CAs are transported outside of the university, the Carriage of dangerous goods regulations should be referred to literature.^15^

## 8. Emergency procedure

There should be an ’out of hours’ procedure, with a spill kit placed next to it, in addition to a risk analysis documentation/Document of mitigation accessible at the laboratory where the CAs are handled.

Researcher should complete “unattended working” form for unattended experiments e.g. LC-MS with clear details of drugs being used, amounts/concentrations and contact details. This form should be displayed next to instrument and another copy lodged with security or equivalent.

Likely emergency or problem scenarios should be assessed and mitigating procedures outlined e.g. power failure, chemical spillage, and evacuation of building due to fire.

In case of any major incident, CARL researchers should be aware of location of phone, fire alarms and power isolation devices. For minor incident (e.g. spillage) CARL researchers should be aware of procedures for dealing with it, contact details of supervisor and logging the incident.

A clearly labelled cytotoxic spill kit should be kept wherever cytotoxic medications are being prepared, stored, or analysed.

## 9. Assurance of compliance

### 9.1. Inspections

A structured inspection should be carried by a team appointed as per the university guidance, for example made up of the departmental safety officer and an interested subject lead.

Inspections at the university should be carried 3 times per year or once per semester, then it should be integrated within the general inspections of the university health and safety.

There should be a reporting protocol that corresponds to the university management structure

#### 9.1.1. Store inspection

The aim of the store inspection is to

- Ensure risk assessments are maintained and in use
- Ensure records are maintained and that CAs are correctly recorded on entry and removal for use
- Ensure that CAs are securely stored
- Ensure knowledge of spill kit use and location
- Condition of transport of CAs within the site

#### 9.1.2. Laboratory inspection

The aim of the laboratory inspection is to

- Ensure Risk assessments are maintained and used
- Verify staff are trained on the manipulation of CAs and equipment and that the training is recorded
- Ensure records of CAs used are maintained (records as COSHH forms kept in laboratories)
- Ensure safe storage of CAs
- Ensure correct PPE used when CAs are in use
- Ensure correct disposal of CA waste

### 9.2. Audits

The audit should be carried infrequently (every 2-3 years)

#### 9.2.1. Audit structure

Receiving relevant safety documentation detailing;

- The safety structure within the organisation and management responsibilities
- Risk assessments controlling the working practices and perceived hazards
- Training strategy for staff and management on responsibilities, working practices and equipment
- The organisation of relevant safety committees
- Communication systems in place to disseminate information

#### 9.2.2. Audit team

- Research team member, preferably the principle investigator or senior team member from area being audited
- Departmental safety officer
- Corporate safety representative
- External auditor either from another organisation or department
- Appoint lead auditor

#### 9.2.3. Methodology

Selected in depth interviews reflecting the horizontal and vertical staffing structure with the aim to verify the documentation received.

Meeting with senior managers to determine the scope and time, date of audit etc. Determine who will be audited and present an agreed timetable.

Select audit team members with specific responsibilities during the audit

The scope may focus on one aspect of work carried out with CAs or look at all aspects ranging from arrival to disposal.

A debrief meeting with senior managers should be held at the end of the audit detailing any immediate and obvious findings followed by a written report. Finally, a second meeting to agree objectives outlined in the audit report with agreed timescales for implementing the audit findings.

The audit should be considered as a constructive process with recommendations for improvement rather than a penalizing system.

#### 9.2.4. Record keeping

An updated list of investigated compounds in each laboratory should be screened by technical/safety officer: Researchers should notify their laboratory manager of the drug handled.

The researcher should have a log of the agents used in the laboratory

#### 9.2.5. Quality assurance

Wipe sample assessment should be performed for proof of compliance; validated sampling and analytical methods should be adapted from reliable sources or determined beforehand.

The wipe sampling strategy depends on the nature, frequency of use, and load of CA handled at the site; a solvent or wetting agent is added to the surface or wiping material and collected for analysis.

Wipe sample analysis is usually performed using gas chromatography (GC), liquid chromatography (LC), high-performance liquid chromatography (HPLC), and ultra-performance liquid chromatography (UPLC); all in combination with mass spectrometry (MS) or tandem mass spectrometry (MS/MS). Adequate PPEs should be donned during sampling of the areas.^16^

In this context, a novel device was recently introduced to the market for qualitatively detecting the presence of hazardous agents on a given surface within few minutes . Currently, kits are available for Methotrexate and Doxorubicin with a lower limit of detection (LLOD) of 0.1 ng ml^−1^ and for cyclophosphamide with an LLOD of 0.5 ng ml^−1^.

Studies are needed to assess the appropriateness, applicability, and sensitivity of this device in real practice.

## Conclusion

These recommendations were developed in order to safeguard and minimize the prospective hazardous consequences on the health of researchers and university personnel in contact with CAs.

Despite the efforts invested to address all aspects of handling CAs throughout its trajectory in university premises, it remains challenging to envisage all related circumstances. Hence, researchers and assigned authorities in universities should use these recommendations while consulting their professional judgement, experience, and surely, national, state, and/or federal regulations.

## Practice implications

This is the first set of consensus recommendations for CAs in academic research settings, however, a significant proportion of the recommendation might apply to the safe handling of other agents with questionable occupational safety. Today, concerns from occupational exposure is expanding beyond hazardous and cytotoxic agents to include antibiotics, and nanoparticles.
